# Correlation between short- and long-term effects of intravitreal ranibizumab therapy on macular edema after branch retinal vein occlusion: a prospective observational study

**DOI:** 10.1186/s12886-017-0485-4

**Published:** 2017-06-13

**Authors:** Yoshiro Minami, Taiji Nagaoka, Akihiro Ishibazawa, Akitoshi Yoshida

**Affiliations:** 1 Department of Ophthalmology, Nayoro City General Hospital, Nishi 7 Minami 8-1, Nayoro, 096-8511 Japan; 2 Department of Ophthalmology, Asahikawa Medical University, Asahikawa, Japan

**Keywords:** Branch retinal vein occlusion, Macular edema, Ranibizumab

## Abstract

**Background:**

The correlation between the short- and long-term effects of intravitreal ranibizumab (IVR) on macular edema after branch retinal vein occlusion (BRVO) remains unclear. We assessed the correlation between the short- and long-term effects of IVR on macular edema after BRVO.

**Methods:**

Twenty-one eyes with macular edema after BRVO were enrolled in this prospective observational study. We measured the foveal thickness (FT) and the best-corrected visual acuity (BCVA) before, 1 day after, and 1 month after IVR (0.5 mg) and then at least every 2 months thereafter until 6 months after the injection. If the macular edema recurred, another injection was administered. The primary endpoint was the change from baseline in the BCVA (ΔVA).

**Results:**

The mean logarithm of the minimum angle of resolution VA improved significantly (*p* = 0.01, *p* < 0.0001, respectively) after 1 day from 0.65 ± 0.28 to 0.51 ± 0.21 (20/89 to 20/63, Snellen equivalent) and after 6 months to 0.29 ± 0.24 (20/39, Snellen equivalent). The mean FT decreased significantly (*p* < 0.0001) after 1 day from 482 ± 85 μm to 349 ± 75 μm and after 6 months to 305 ± 84 μm. The 1-day VA was significantly (*r* = 0.68, *p* = 0.0007) positively correlated with the 6-month VA. The 1-day ΔVA was significantly (*r* = 0.79, *p* < 0.0001) positively correlated with the 6-month ΔVA.

**Conclusions:**

The short-term effects of IVR may predict the long-term effects of IVR in macular edema secondary to BRVO.

**Trial registration:**

Trial registration number: UMIN000027003. Retrospectively registered. (April/15/2017)

## Background

Retinal vein occlusion (RVO), including branch RVO (BRVO), hemiretinal vein occlusion, and central RVO, and diabetic retinopathy are common retinal vascular diseases that cause moderate or severe visual loss in working-age patients [1–3]. RVOs have been estimated to develop in about 16 million people worldwide, and BRVO accounts for nearly 80% of those cases. The predominant cause of decreased visual acuity (VA) due to RVOs is macular edema when the fovea is involved [1–3]. Because the Branch Vein Occlusion Study Group reported the efficacy of grid laser treatment for macular edema after BRVO, it has been the standard therapy for treating macular edema associated with BRVO for about 30 years [4].

The Ranibizumab for the Treatment of Macular Edema following Branch Retinal Vein Occlusion: Evaluation of Efficacy and Safety (BRAVO) study found that ranibizumab (Lucentis, Novartis, Basel, Switzerland), a humanized affinity-matured vascular endothelial growth factor (VEGF) antibody fragment that specifically binds all isoforms of VEGF-A, clearly improved macular edema due to BRVO [5, 6]. We recently reported the short-term effect of intravitreal ranibizumab (IVR) injections on macular edema secondary to BRVO, and the short-term effect (within a day) was correlated with the therapeutic efficacy 1 week and 1 month after the IVR injection [7]. However, the correlation between the short- and long-term effects of IVR injections on macular edema due to BRVO is undetermined. In the current study, we report the short-term (1 day) and the long-term (6 months) effects and the correlation between them.

## Methods

### Subjects

The study adhered to the tenets of the Declaration of Helsinki and followed the guidelines approved by the ethics committee of our institution. All patients were treatment-native Japanese individuals who provided informed consent before participation in the study. The study was performed at Nayoro City General Hospital between October 2013 and April 2016. The inclusion criteria were the presence of macular edema that involved the fovea after development of BRVO before therapy (foveal thickness [FT] at baseline ≧ 300 μm), no history of IVR injection, and no history of other treatments for macular edema within the previous 3 months. Application of photocoagulation to the unvascularized area outside of the vessel arcade before and throughout the follow-up period was permitted. No patients were treated with a dexamethasone implant because the treatment had not yet been approved in Japan. The exclusion criteria were the presence of other retinal diseases such as diabetic retinopathy and age-related macular degeneration. Patients diagnosed with hemicentral vein occlusion were considered to have BRVO according to the BRAVO study criteria [5]. The patients underwent comprehensive ophthalmologic examinations including measurement of the best-corrected visual acuity (BCVA), slit-lamp biomicroscopy with a noncontact fundus lens, measurement of intraocular pressure (IOP) with noncontact tonometers, and spectral-domain optical coherence tomography (SD-OCT) (RetinaScan RS-3000, Nidek, Gamagori, Japan). The BCVA was measured using a standard Japanese decimal VA chart at 5 m. The decimal values were converted to the logarithm of the minimum angle of resolution (logMAR) units for statistical analyses. To evaluate the FT, the macular map analysis protocol of the RS-3000 SD-OCT was used. The FT was defined as the average of all points in the inner circle (radius, 1 mm) at the center of the nine sectors defined by the Early Treatment Diabetic Retinopathy Study grid [8].

### IVR injection

IVR injections were administered according to a sterile technique (0.5 mg/0.05 mL) using a 30-gauge needle. Before injection, anterior chamber paracentesis was performed using a 27-gauge needle to prevent IOP increases. Topical antibiotics were instilled prophylactically for 1 week after the IVR injection.

#### Time course of evaluation of the therapeutic effect of IVR injections and criteria for additional injections

The FT and BCVA were measured before the IVR injection (baseline) and 1 day after, 1 month after, and at least every 2 months (1–2 months) after the IVR injection until 6 months. If recurrent macular edema was detected, another injection was administered. Recurrent macular edema was defined as an increase in the FT exceeding 50 μm over a previous measurement. Patients who did not present at the time of examination or did not receive another injection despite recurrent macular edema were excluded from the current study. The changes in the logMAR VA (ΔVA) from baseline were calculated as ΔVA-1 day and −1, −3, and −6 months. The changes in the FT (ΔFT) from baseline also were calculated as ΔFT-1 day, −1, −3, and −6 months.

### Data analysis

All values are expressed as the means ± standard deviations. Overall differences in the logMAR VA and the FT between baseline and 1 day and 1, 3, and 6 months after the IVR injections were assessed using repeated measures analysis of variance. Subgroup-specific changes in the VA and FT were analyzed using Dunnett’s multiple comparisons test. To determine if the early effectiveness of IVR injections predicts the late-phase outcome after the injections, the correlations between the VA at 1 day and the VA at 6 months and between the ΔVA-1 day and the ΔVA-6 months and between the ΔFT-1 day and the ΔVA-6 months were evaluated using Pearson’s correlation model and linear regression analysis. To investigate the correlations between the symptom duration and the VA at 6 months or the ΔVA-6 months, Spearman’s rank correlation coefficient was used because the symptom duration was wide. *P* < 0.05 was considered significant.

## Results

Table 1 shows the patient baseline characteristics. Thirty-five consecutive eyes of 34 patients with macular edema due to BRVO who received the first IVR injection during this study were included. Fourteen eyes of 13 patients were lost to follow-up during the study. Thus, 21 eyes of 21 patients were analyzed. The mean number of injections was 2.0 ± 0.59 during this study. Two eyes underwent photocoagulation before study entry and two eyes underwent photocoagulation of non-perfused areas outside of the vessel arcade during this study. Eleven patients had systemic hypertension and no patients had diabetes mellitus. Two eyes had an intraocular lens implanted previously. The average time from the development of BRVO to the IVR injection was 9.4 ± 11.2 months. Ocular hypertension (>21 mmHg) or a drastic IOP elevation (elevation more than 5 mmHg from baseline) did not occur at any time points. No treatment complications such as endophthalmitis or retinal detachment developed during the study.

**Table 1:** Baseline Characteristics of 21 Patients (21 Eyes) with Macular Edema after BRVO

Age (years, mean ± SD)	71.0 ± 10.3
Gender (male/female)	6/15
Symptom duration (months, mean ± SD)	9.4 ± 11.2
History of photocoagulation (eyes)	2 (10%)
BCVA (logMAR, mean ± SD) (Snellen equivalent)	0.65 ± 0.28 20/89
Baseline FT (μm, mean ± SD)	482 ± 85
Phakic eye (eyes)	19 (90%)
Systemic hypertension (patients)	11 (52%)
Diabetes mellitus (patients)	0 (0%)

*BRVO* branch retinal vein occlusion, *logMAR* logarithm of the minimum angle of resolution, *SD* standard deviation, *BCVA* best-corrected visual acuity, *FT* foveal thickness

**Fig. 1:**
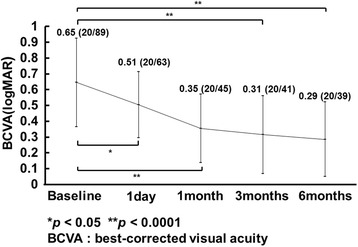
The mean changes in the logarithm of the minimum angle of resolution (logMAR) best-corrected visual acuity (BCVA) at each follow-up evaluation (*n* = 21 eyes). The values are expressed as the mean ± standard deviation (Snellen equivalent). The BCVA is improved significantly from baseline (*p < 0.05, **p < 0.0001)

**Fig. 2:**
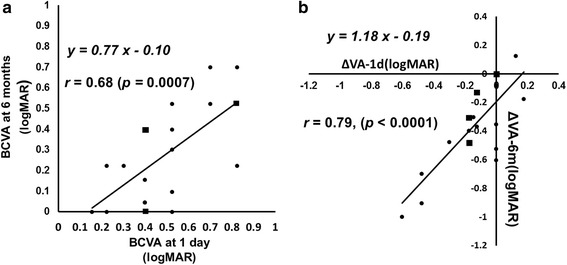
**a** The relationship between the changes in the logarithm of the minimum angle of resolution (logMAR) best-corrected visual acuity (BCVA) 1 day after the first intravitreal ranibizumab (IVR) injection and the BCVA 6 months after the first IVR injection. There is a significant (*r* = 0.68, *p* = 0.0007) positive correlation between them. The squares indicate that two eyes overlap. **b** The relationship between the changes in the logMAR BCVA from baseline to 1 day (ΔVA-1 day) and the changes in the BCVA from baseline to 6 months (ΔVA-6 months) after the IVR injections. There is a significant (*r* = 0.79, *p* < 0.0001) positive correlation between them. The squares indicate that two eyes overlap

**Fig. 3:**
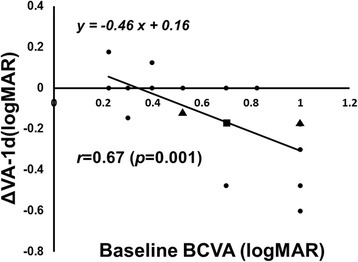
The relationship between the changes in the logarithm of the minimum angle of resolution best-corrected visual acuity (logMAR BCVA) from baseline to 1 day (ΔVA-1 day) and the baseline VA. There is a significant (*r* = 0.67, *p* = 0.001) positive correlation between them. The squares indicate that two eyes overlap; the triangles indicate that three eyes overlap

Figure 1 shows the mean changes in the logMAR VA after the IVR injection. The mean logMAR VA improved significantly on 1 day and 1, 3, and 6 months (*p* = 0.01, *p* < 0.0001, *p* < 0.0001, and *p* < 0.0001, respectively) from the baseline values of 0.65 ± 0.28 (20/89, Snellen equivalent) to 0.51 ± 0.21 (20/63 Snellen equivalent), 0.35 ± 0.22 (20/45, Snellen equivalent), 0.31 ± 0.25 (20/41, Snellen equivalent), and 0.29 ± 0.24 (20/39, Snellen equivalent) after the IVR injections. The mean FT decreased significantly (*p* < 0.0001) from the baseline value of 482 ± 85 μm to 349 ± 75 μm 1 day after the IVR injection. Significant reductions also were seen to 263 ± 39, 319 ± 96, and 305 ± 84 μm at 1, 3, and 6 months, respectively (*p* < 0.0001 at all time points). The BCVA 1 day after the IVR injection was correlated significantly (*r* = 0.68, *p =* 0.0007) with the BCVA 6 months after the IVR injection (Fig. 2a). The ΔVA-1 day was correlated significantly (*r* = 0.79, *p* < 0.0001) with the ΔVA-6 months (Fig. 2b). The ΔFT-1 day was not correlated significantly (*r* = 0.09, *p* = 0.68) with the ΔVA-6 months. The symptom duration was correlated significantly (*p* = 0.003) with the BCVA 6 months after the IVR injection but not with the ΔVA-6 months (*p* = 0.12). The ΔVA-1 day was correlated significantly (*r* = 0.67, *p* = 0.001) with the baseline VA (Fig. 3).

## Discussion

In the current study, the BCVA and FT improved significantly 1 day after the IVR injection from the baseline values, and those improvements were sustained 6 months after the first IVR injection (Fig. 1). We recently reported that the BCVA and FT improved significantly from the baseline values within 1 day after the IVR injection in patients with macular edema secondary to BRVO [7]. In the current study, we showed that the BCVA 1 day after the IVR injection was correlated with the BCVA 6 months after the IVR injection in patients with macular edema due to BRVO (Fig. 2a) and the ΔVA-1 day was correlated more significantly with the ΔVA-6 months (Fig. 2b). This indicated that we can predict the long-term (6-month) functional outcome based on the short-term (within 1 day) functional effect of the IVR injections. In contrast, the ΔFT-1 day was not correlated significantly with the ΔVA-6 months, suggesting that it is difficult to predict the long-term functional effect based on the short-term structural change after the IVR injections. Although the baseline BCVA was reported to be predictive of the long-term effect of IVR injections [9], the baseline BCVA was not correlated significantly (*p* = 0.09) with the BCVA 6 months after the first IVR injection in the current study. However, in the current study, the BCVA 1 day after the IVR injection was correlated significantly with the BCVA at 6 months after the IVR injection and the ΔVA-1 day was correlated significantly with the ΔVA-6 months (Fig. 2b). Our results suggested that measuring the BCVA 1 day after the IVR injection might be a better predictive factor of the long-term effect of IVR injections on the macular edema secondary to BRVO.

The symptom duration has been reported to be predictive of the efficacy of anti-VEGF therapy on the macular edema secondary to BRVO [9, 10]. In the current study, the symptom duration was correlated significantly with the BCVA 6 months after the first IVR injection. However, the symptom duration was not correlated significantly with the baseline BCVA. The current results suggested that we can obtain better long-term effects of the IVR injections when the first injection is administered sooner after the onset of BRVO.

Although the result might be affected by the protocol for the therapy, the best protocol for an IVR injection to treat BRVO remains controversial, i.e., monthly consecutive injections (mostly for 3 months) or pro re nata (PRN) injections. As reported in the BRAVO study [5, 6] and other clinical studies [11, 12], consecutive IVR injections are effective for treating the macular edema associated with BRVO. However, several authors have reported the efficacy of PRN intravitreal bevacizumab (IVB) (Avastin, Genentech Inc., South San Francisco, CA) and IVR injections [9, 10, 13, 14]. Ito et al. [15] reported that the group treated with PRN IVB injections for macular edema secondary to BRVO achieved similar visual outcomes with fewer injections compared with the group treated with three consecutive IVB injections. Therefore, it is important to achieve an effectiveness level similar to that of IVR injections with fewer injections, and we believe that PRN injections are the best protocol for IVR therapy to treat macular edema secondary to BRVO. Therefore, in the current study, we followed a PRN injection protocol.

The current study had some limitations. First, the number of patients was too small for subgroup analysis. Because the prognosis for the VA recovery was reported to be affected by the duration of the macular edema and previous treatment before the IVB injection [10], we excluded patients who had been treated previously for macular edema within 3 months of study entry. We previously reported that the BCVA and FT improved significantly from the baseline within 1 day after the IVR injection in patients with macular edema secondary to BRVO in 23 eyes [7]. Moreover, we also revealed that the ΔVA-1 day was correlated significantly with the ΔVA-1 month. Although the number of eyes was small, the current result regarding the relationship between the ΔVA-1 day and ΔVA-1 month was similar to our previous report [7]. Second, because the current study had no control group, we could not exclude the effect of spontaneous regression on the effect of the IVR injections on macular edema. Macular edema secondary to BRVO has a high level of spontaneous regression within 3 months of the occurrence [4]. We could not exclude the effect of spontaneous regression on our results. Indeed, seven current patients received an IVR injection less than 3 months after the occurrence of BRVO. We confirmed that the current results (significant correlation between VA at 1 day and VA at 6 months after the IVR injection and between the ΔVA-1 day and ΔVA-6 months) were unchanged after the data from those seven patients were removed from the analysis (data not shown). Taken together, we speculated that the effect of spontaneous regression had little effect on the current results. In addition, previous major clinical studies have included patients in whom macular edema developed within 3 months before the first IVR injection.

## Conclusion

In conclusion, the current findings suggested that the effects of IVR injections might be detectable as early as 1 day after treatment and evaluation of the short-term effects of IVR injections can predict the long-term outcome of IVR injections administered to treat macular edema secondary to BRVO.

## Abbreviations

BCVA: Best-corrected VA
BRVO: Branch retinal vein occlusion
FT: Foveal thickness
IVB: Intravitreal injection of bevacizumab
IVR: Intravitreal ranibizumab
logMAR: Logarithm of the minimum angle of resolution
PRN: Pro re nata
RVO: Retinal vein occlusion
SD-OCT: Spectral-domain optical coherence tomography
VA: Visual acuity
VEGF: Vascular endothelial growth factor
ΔFT: Changes in the FT
ΔVA: Changes in the logMAR VA
